# Avian pathogenic *Escherichia coli* (APEC) infection alters bone marrow transcriptome in chickens

**DOI:** 10.1186/s12864-015-1850-4

**Published:** 2015-09-15

**Authors:** Hongyan Sun, Peng Liu, Lisa K. Nolan, Susan J. Lamont

**Affiliations:** Department of Animal Science, Iowa State University, Ames, Iowa 50011 USA; Department of Statistics, Iowa State University, Ames, Iowa 50011 USA; Department of Veterinary Microbiology and Preventive Medicine, Iowa State University, Ames, Iowa 50011 USA

**Keywords:** RNAseq, APEC, Bone marrow, Transcriptome, Immune response

## Abstract

**Background:**

Avian pathogenic *Escherichia coli* (APEC) is a major cause of disease impacting animal health. The bone marrow is the reservoir of immature immune cells; however, it has not been examined to date for gene expression related to developmental changes (cell differentiation, maturation, programming) after APEC infection. Here, we study gene expression in the bone marrow between infected and non-infected animals, and between infected animals with mild (resistant) versus severe (susceptible) pathology, at two times post-infection.

**Results:**

We sequenced 24 bone marrow RNA libraries generated from the six different treatment groups with four replicates each, and obtained an average of 22 million single-end, 100-bp reads per library. Genes were detected as differentially expressed (DE) between APEC treatments (mild pathology, severe pathology, and mock-challenged) at a given time point, or DE between 1 and 5 days post-infection (dpi) within the same treatment group. Results demonstrate that many immune cells, genes and related pathways are key contributors to the different responses to APEC infection between susceptible and resistant birds and between susceptible and non-challenged birds, at both times post-infection. In susceptible birds, lymphocyte differentiation, proliferation, and maturation were greatly impaired, while the innate and adaptive immune responses, including dendritic cells, monocytes and killer cell activity, TLR- and NOD-like receptor signaling, as well as T helper cells and many cytokine activities, were markedly enhanced. The resistant birds’ immune system, however, was similar to that of non-challenged birds.

**Conclusion:**

The DE genes in the immune cells and identified signaling models are representative of activation and resolution of infection in susceptible birds at both post-infection days. These novel results characterizing transcriptomic response to APEC infection reveal that there is combinatorial activity of multiple genes controlling myeloid cells, and B and T cell lymphopoiesis, as well as immune responses occurring in the bone marrow in these early stages of response to infection.

**Electronic supplementary material:**

The online version of this article (doi:10.1186/s12864-015-1850-4) contains supplementary material, which is available to authorized users.

## Background

Avian pathogenic *Escherichia coli* (APEC) can cause colibacillosis due to immunosuppression and damage of the immune system [1, 2]. Infection is typically initiated in the respiratory tract by inhalation of fecal dust from which it can gain access to the bloodstream and immune organs, causing septicemia, pericarditis, and mortality [3, 4]. Similar phylogenic backgrounds and certain virulence genes are present in both APEC and human extra-intestinal pathogenic *Escherichia coli* (ExPEC) [5]. Thus, poultry products contaminated with APEC are a potential source of foodborne ExPEC infection to humans, posing a threat to human health [6–9]. Although vaccination offers one route to control APEC, many vaccines may only be effective against homologous APEC challenge [10, 11]. Consequently, a more comprehensive understanding of chicken responses to APEC will facilitate the improvement of control strategies, vaccine development, and human health.

Bone marrow, the source of pluripotent hematopoietic stem cells, is a reservoir for two main categories of white blood cells, the lymphoid and myeloid lineages [12]. The lymphoid lineage differentiates into B, T, and natural killer cells, while the myeloid lineage develops into macrophages, granulocytes, mast cells, and dendritic cells (DCs) [12–14]. All these cells play critical roles in innate and adaptive immune responses. Genome-wide gene expression profiling of immune organs or cells has become a major method to simultaneously compare the expression levels of hundreds of thousands of genes between different conditions [15–18].

Bone marrow is an excellent tissue source for genomic scale gene expression profiling in APEC infection because it provides primordial cells that have not been influenced by developmental cytokines and other factors that would be present in the lymphoid organs. The study of bone marrow, therefore, offers new avenues to elucidate a comprehensive picture of the immune mechanism the primary lymphoid organ depends on to respond to APEC infection at the early transcriptional level. Here, we study gene expression in the bone marrow between infected and non-infected animals, and between infected animals with mild (resistant) vs. severe (susceptible) pathology, at two times post-infection.

## Methods

### Ethics statement

All animal care and experimental procedures were reviewed and approved by the Animal Care and Use Committee (#11-07-6460-G) of the Research Animal Resources Center at Iowa State University.

### Experimental design

**Fig. 1:**
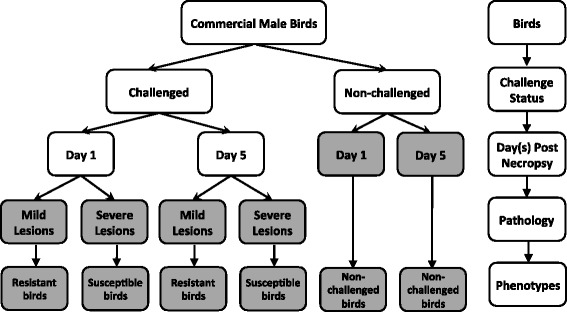
Experimental design. Chicks were divided into each of three different conditions: APEC challenge status, tissue harvest time, and pathology level. Based on the severity of lesions in liver, pericardium, and air-sac scored at necropsy, the challenged birds were assigned to either mild or severe pathology categories, representing resistant and susceptible phenotypes, respectively. Birds in six treatments total were studied: day 1 resistant, day 1 susceptible, day 5 resistant, day 5 susceptible, day 1 non-infected, and day 5 non-infected

A total of 360 commercial broiler male chicks, obtained at day of hatch from the same commercial supplier, were studied in six replicated experiments. For each replicate, 48 male meat-type (broiler) chickens at 4 weeks of age were challenged with 0.1 ml APEC O1 introduced by the intra-air sac route, and 12 birds in a control group were mock-challenged with 0.1 ml of phosphate buffered saline (PBS). Birds were euthanized and bone marrow was harvested at 1 and 5 days post-infection (dpi), from half of the birds at each time point. The sample collection times were chosen to include the day of maximum symptoms (5 dpi) based on previous studies of co-author Nolan’s group with the same bacteria [9] and one early day (1 dpi) to assess the immediate, early changes in gene expression occurring in the tissues. A veterinary pathologist visually inspected and scored the severity of the lesions in three tissues - air sacs, pericardium, and liver - according to the standard pathology scoring system described by Peighambari et al. [19]. Scores for pericardium and liver were 0–2, for air sacs were 0–3. Birds with summed lesion scores of 0–3 were classified as the “mild” (resistant) infected group and those with summed scores of 4–7 were classified as the “severe” (susceptible) infected group. Birds with intermediate pathology scores were not included in the transcriptome study. In total, six treatments resulted across challenge status, day post-infection necropsy, and pathology level (Fig. 1)

. The APEC O1 strain is highly virulent in birds and its genome has been completely sequenced [9]. Detailed information on the APEC O1 strain and related procedures have been previously published [20, 21].

### Total RNA extraction

Four birds (one from each of four replicates) from each of the six conditions were selected, resulting in 24 samples used for RNAseq. The RNA samples were isolated using the Ambion MagMax-96 Kit (AM1839) (Applied Biosystems, Foster City, CA) and immediately stored at −80 °C. All extraction procedures were performed according to manufacturer’s recommendations. A NanoDrop ND-1000 UV–vis Spectrophotometer (NanoDrop Technologies Inc., Wilmington, DE, USA) was used to quantify the RNA. In addition, the quality of RNA was tested using the Agilent 2100 Bioanalyser (Agilent Technologies). The RNA Integrity Number (RIN) was greater than 9.0 for all samples.

### cDNA library construction and illumina deep sequencing

The initial total RNA was converted into a cDNA sequencing library through the Illumina TruSeq® RNA Sample Preparation v2 Kit following the manufacturer’s instructions (Protocol: #15026495, May 2012). First, 0.1 - 4 g total RNA was purified using Poly-T oligo-attached magnetic beads to obtain the mRNA fragments. Next, the first-strand cDNA was synthesized from the mRNA fragments. Finally, second-strand cDNA synthesis, end repair, 3’ end adenylation, and adapter ligation were performed and PCR amplification was conducted. The cDNA libraries were validated and quantified using a Qubit® Quantitation Platform and an HS dsDNA kit (Invitrogen, Paisley, UK). The 24 cDNA libraries were individually generated from 24 samples. One library from each of the six treatments was pooled into one lane to sequence. Thus, four lanes were used to sequence the 24 cDNA libraries through the Illumina® HiSeq 2000 at the Iowa State University (ISU) DNA facility.

The image files were converted into sequences using Illumina Software to obtain 100 bp single-end reads during sequencing of fragment clusters.

### RNAseq analysis

For each of the sequencing reads, low-quality bases (Sanger base quality < 20) of 3’ ends were first trimmed using in-house perl scripts and the sequencing adapters were then trimmed using Fastx toolkit (version 0.0.13) software. Quality of the reads was determined by FastQC software (version 0.10.1). All Illumina single-end reads of 24 samples from six treatments were mapped separately to Ensembl *Gallus gallus* 4.0 reference genome by TopHat software (version 2.0.9) and Bowtie (version 2.1.0) using default parameters. The HTseq package (version 0.5.4p3) in Python was used to calculate the number of aligned reads per exon through Ensembl annotation of the chicken genome. The number of read counts per gene was identified in the output file by Ensembl gene ID. The RNAseq data can be obtained from the Gene Expression Omnibus (GEO) database with the accession number GSE67302.

Qlucore Omics Explorer (v3.0) was used to perform principle component analysis (PCA). The detected genes (count number > 1) from the 24 samples were *log*_2_ transformed and subjected to normalization (mean = 0 and variance = 1). The Bioconductor package edgeR (version 3.0.8) developed in R software (version 2.15.3) was used to identify the differentially expressed (DE) genes. Trimmed mean of M-values (TMM) normalization method [22] was performed in edgeR to normalize the data across libraries. Generalized linear models based on negative binomial distributions were fit to the data using edgeR, and the model includes treatment and replicate effects. The Benjamini-Hochberg (BH) method was applied to control the false discovery rate (FDR) [23]. A gene was designated DE if its fold change of expression was above 1.5 while FDR was controlled at 5 %.

Cell type enrichment (CTen) was used to analyze the cell-type specificity for the RNAseq data to detect changes in the cellular demographics [24]. The cell type enrichment analysis was measured by the enrichment score, the –log_10_ of the BH adjusted *p* values [25]. The GOseq package (version 1.10.0) was used to detect the enrichment of gene ontologies (GO) among the list of DE genes. For heatmap pathway analysis, bayesian likelihood ratio test was used to determine the goodness-of-fit. Then, the ratio was transformed to a z-score test statistic (Bayesian z-score) to permit comparison of scores across all pathways.

### Quantitative PCR (qPCR) and statistical analysis

Fifteen genes, ADA, BLNK, CD3D, CD28, CD40, CD3Z, IFNG, IL1, IL7, IL8, IL18, LIG4, MD2, NOD1, TLR4, were selected to confirm RNAseq results. Criteria for gene selection were based on immune response function and significance in the RNAseq study. 28S, a housekeeping gene, was utilized to normalize the starting concentration of RNA. Primer sequences for the fifteen selected genes were designed by using sequences from NCBI and PRIMER3 [26]. Primer sequence details are provided in Additional file 1: Table S1. All genes assays were run in triplicate for the same individual samples as the RNAseq. qPCR was conducted using QuantiTect SYBR Green RT-PCR kit (Qiagen Inc., Valencia, CA). The adjusted cycle threshold (Ct) values were calculated using the equation: 40 – [Ct sample gene mean + (Ct 28S median – Ct 28S mean)(slope of sample gene/slope of 28S)]. The qPCR data were analyzed using JMP 8.0.2 statistical software (SAS Institute Inc., Cary, NC). The mRNA expression levels were measured as the mean adjusted Ct values of each triplicate sample. The analysis model used in the ANOVA analysis of JMP 8.0.2 was as following: Y = *μ* + challenge + day collection + replicate + e. Challenge, day collection, and replicate were considered as fixed effects. e was the random effect. Student’s t test of JMP 8.0.2 was performed to test the significant difference between different contrasts. The significance level was set at 0.05. Fold change was measured by the equation: 2^(adjusted Ct value of treatment A – adjusted Ct value of treatment B)^. Gene expression fold change and significance in qPCR were used to compare with RNAseq for different contrasts.

## Results

### Transcriptome sequence

Twenty-four individual samples (4 different individuals of 6 treatments) were analyzed by RNAseq. RNA sequencing resulted in 11 to 40 million single-end raw reads of 100 bp per sample (Table 1)

**Table 1:** Summary of numbers of sequencing reads, mapped reads, mapping rate, detected genes and transcriptome coverage

Treatments			Raw reads	Mapped reads	% of reads Mapped	Detected genes	Transcriptome coverage
dpi	Phenotype	Bird #					
1	Non-challenged	1	40,958,949	32,154,546	80.3 %	14,986	87.59 %
1	Non-challenged	2	17,774,235	13,758,514	78.7 %	14,275	83.44 %
1	Non-challenged	3	17,409,364	13,812,510	80.7 %	14,140	82.65 %
1	Non-challenged	4	38,557,156	26,754,843	70.6 %	14,859	86.85 %
1	Resistant	1	21,110,683	15,919,755	78.6 %	14,432	84.36 %
1	Resistant	2	19,457,693	14,734,512	79.8 %	14,374	84.02 %
1	Resistant	3	31,249,434	24,654,203	80.4 %	14,936	87.30 %
1	Resistant	4	26,426,437	21,574,698	83.0 %	14,640	85.57 %
1	Susceptible	1	26,940,892	20,423,349	79.3 %	14,759	86.27 %
1	Susceptible	2	16,227,837	11,585,446	79.1 %	13,954	81.56 %
1	Susceptible	3	23,554,069	18,481,538	79.8 %	14,713	86.00 %
1	Susceptible	4	19,045,548	15,352,573	82.2 %	14,409	84.22 %
5	Non-challenged	1	40,113,450	31,164,137	79.5 %	14,966	87.48 %
5	Non-challenged	2	15,799,657	12,039,801	80.0 %	13,947	81.52 %
5	Non-challenged	3	20,791,304	16,735,997	81.8 %	14,235	83.21 %
5	Non-challenged	4	27,189,817	21,665,457	81.1 %	14,553	84.95 %
5	Resistant	1	20,286,505	14,862,432	79.8 %	14,296	83.56 %
5	Resistant	2	11,761,420	8,652,658	80.0 %	13,543	79.16 %
5	Resistant	3	18,361,293	14,341,042	80.2 %	14,281	83.48 %
5	Resistant	4	16,590,846	13,386,031	80.7 %	14,038	82.06 %
5	Susceptible	1	22,585,434	17,629,308	80.7 %	14,467	84.56 %
5	Susceptible	2	11,789,716	8,534,110	78.7 %	13,839	80.89 %
5	Susceptible	3	17,874,004	13,601,653	77.9 %	14,495	84.73 %
5	Susceptible	4	15,429,659	12,406,170	82.0 %	14,163	82.79 %

Note: dpi: day post-infection

. After alignment, an average of 80 % of the reads, with 5 % representing multiple mapping, were mapped to the chicken reference transcriptome (Table 1). There are 17,108 annotated chicken genes in the Ensembl database (http://useast.ensembl.org/biomart/martview/1744c16506e92aabbac0907509c58539). On average, 14,388 chicken genes were detected for an individual, accounting for about 84 % of all 17,108 annotated chicken genes in the database (Table 1). Among these detected unique genes, there were 2,404 novel genes, mainly on chromosomes 1, 2, 4, 3, Z, and 5 in decreasing number. A total of 9,569 genes were included in the statistical analysis by retaining only genes that were detected with the read count above 2 counts per million for each sample in at least one treatment group.

### Pathology level and sample similarity

**Fig. 2:**
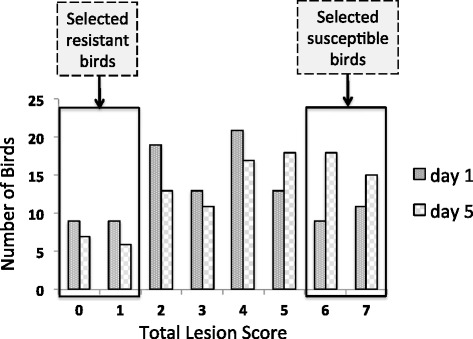
Summation of lesion score distribution of the challenged birds at both times . Transcriptomes were studied of birds lesion scores of 0–1 (resistant) and 6–7 (susceptible). The average lesion scores of the four replicates resistant and susceptible birds is 0.375 and 6.625, respectively

After infection, the birds exhibited normally distributed summed pathology scores (Fig. 2)

**Fig. 3:**
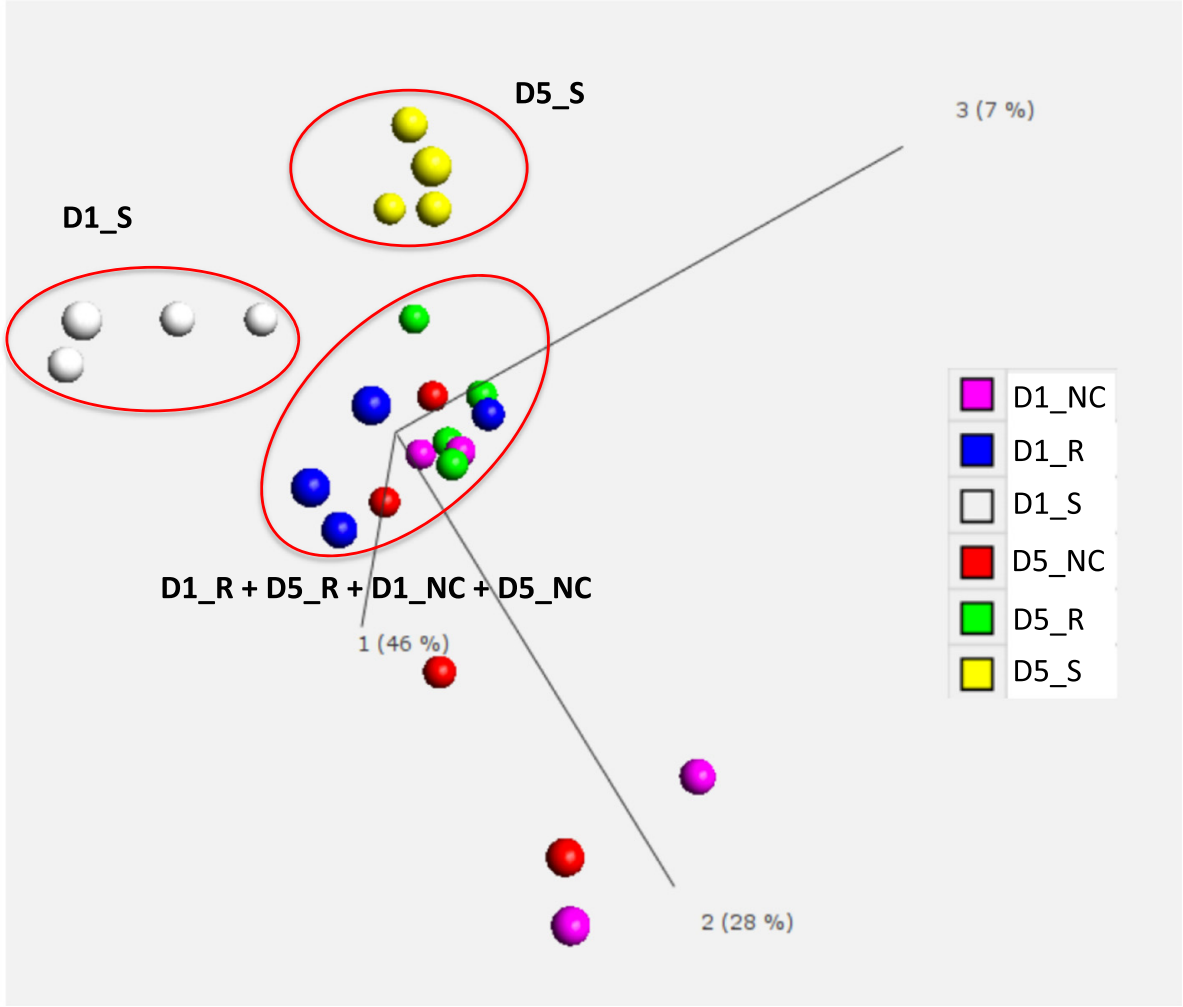
Principal component analysis (PCA) of transcriptome of all 24 RNAseq libraries. Across the complete dataset, 20.46 % (3501/17108) genes were differentially expressed with the false discovery rate (FDR) < 0.05 and fold change > 1.5. Each point indicates one RNAseq library. The susceptible birds at the two time points clustered separately. The other four treatments, however, were clustered together. Abbreviations: D1_NC, day 1 non-challenged birds; D1_R, day 1 resistant birds; D1_S, day 1 susceptible birds; D5_NC, day 5 non-challenged birds; D5_R, day 5 resistant birds; D5_S, day 5 susceptible birds

. For the RNAseq, we used birds of high and low pathology scores, to represent disease-susceptible and disease-resistant phenotypes, respectively. Birds with 0 to1 lesion score (average 0.375) as resistant phenotype, and birds with 6 to 7 lesion score (average 6.625) as the susceptible phenotype were used. For the RNAseq dataset, PCA was used to identify sample similarity among the six treatments (Fig. 3)

. The susceptible birds (severe pathology) at both time points were separately clustered. The other four treatments were intermingled in the PCA. Results clearly indicate that challenged-susceptible birds exhibit transcriptomic changes that are distinct from the challenged-resistant and non-challenged birds, and that this is also influenced by time post-infection. However, there is very little difference in transcriptomes between challenged-resistant and non-challenged birds.

### DE genes in bone marrow

**Fig. 4:**
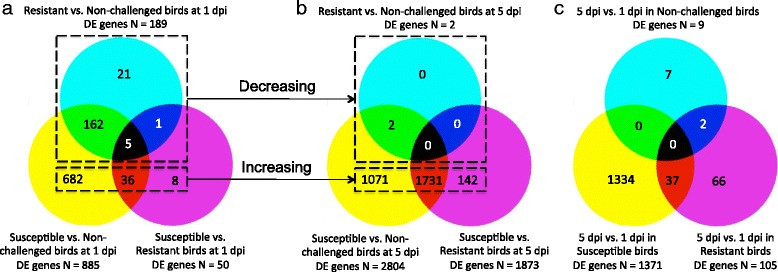
Comparison of the identified differentially expressed (DE) genes across time and treatment (challenge and pathology status) effects. The number in overlapped regions is the number of DE genes that were detected in all three contrasts or each two contrasts. **a** Represents shared and unique significant genes for treatment effect at 1 day post-infection (dpi). **b** Indicates shared and unique significant genes for treatment effect at 5 dpi. **c** Shows shared and unique DE genes for time effect post-challenge. **a** and **b** demonstrate that the number of DE genes in resistant vs. non-infected birds were decreasing over the two days, while the number of DE genes in susceptible vs. non-infected and susceptible vs. resistant birds increased with time post-infection

Differences in treatment groups associated with pathology were detected by contrasts of treatment groups within each time point, while differences associated with time effects were detected by contrasts of the two time points within each treatment group. Consequently, nine total contrasts were generated. Figure 4 shows the numbers of shared and unique DE genes based on treatment effects within time and time effects within treatment.

At 1 dpi, in comparison to non-challenged birds, hundreds of DE genes were detected in resistant birds (N = 189) and in susceptible birds (N = 885). The numbers of up-regulated DE genes were far greater than the numbers down-regulated. At 1 dpi, only 5 significantly DE genes were co-expressed in three of the contrasts (Fig. 4a)

. There were 162 DE genes shared between susceptible vs. resistant birds and susceptible vs. non-challenged birds on day 1, while only small numbers of DE genes were shared in other contrasts (Fig. 4a). These results suggest that early after infection, 1 dpi, the bone marrow gene expression of challenged-susceptible birds was similar to that of challenged-resistant birds, and that challenged birds differed significantly from non-challenged birds.

At 5 dpi, the bone marrow transcriptome of resistant birds was very similar to non-challenged birds, with only two genes detected as DE between the two conditions (Fig. 4b). Between the contrasts of susceptible vs. non-challenged birds and of susceptible vs. resistant birds, 59 % (1,371/2,946) of DE genes were in common. There were 1071 and 142 unique DE genes in susceptible vs. non-challenged birds and susceptible vs. resistant birds, respectively, on day 5 (Fig. 4b). Generally, the numbers of DE genes in resistant vs. non-challenged birds decreased over time, while the numbers of DE genes increased over time in the susceptible vs. non-challenged and the susceptible vs. resistant contrasts (Fig. 4a and b). These results suggest that the bone marrow transcriptome of challenged-resistant birds was returning to a homeostatic state by 5 dpi. Challenged-susceptible birds continued to diverge from both challenged-resistant and non-challenged birds as post-challenge time progressed from 1 dpi to 5 dpi.

There was little difference (N = 9) in the bone marrow transcriptome of non-challenged birds at 5 vs. 1 dpi. However, both infected treatment groups (resistant and susceptible) had large numbers of DE genes (N = 105; N = 1371 respectively) over time, especially the susceptible birds (Fig. 4c). Of the 1,371 DE genes, 97 % (1,334) of them were unique in susceptible birds. These results demonstrate that challenged-susceptible birds have a unique gene expression profile that diverges over time from that of challenged-resistant and non-challenged birds.

### DE gene cell specific activity and GO terms assignments

**Fig. 5:**
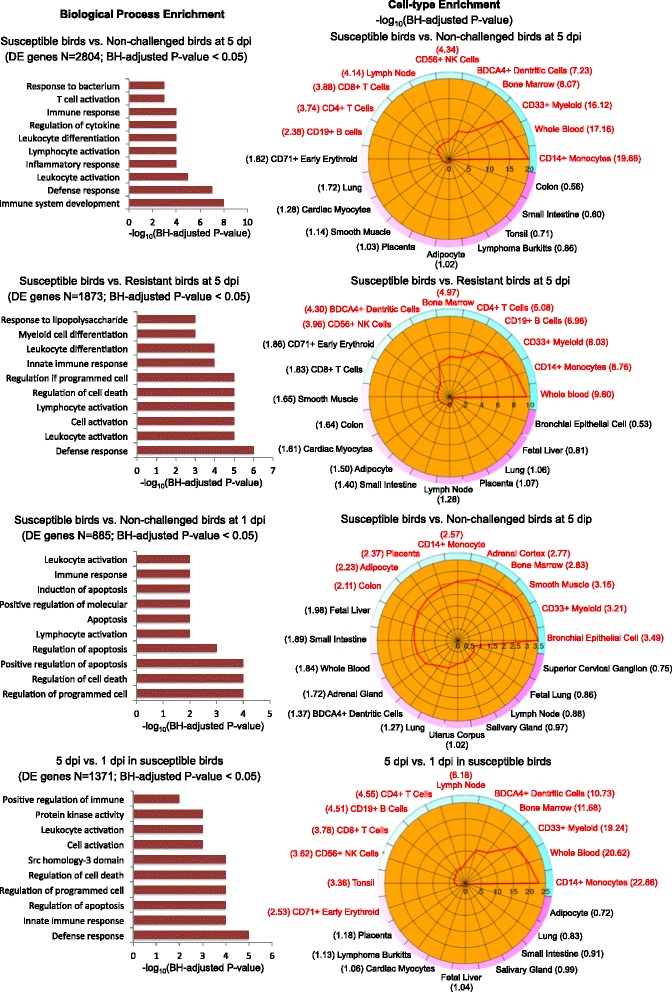
GO terms and CTen analysis. The list of differentially expressed (DE) genes in bone marrow from broilers infected with APEC is analyzed by GOseq and CTen. The left shows the functional annotation for DE genes in the four contrasts: top ten significant GO biological processes. The right shows the summary of cell type enrichment analysis expressed as –log10(Benjamini and Hochberg adjusted P value). The characters in red color indicate significantly enriched cell type. The color from blue to pink indicates the enrichment from the highest to lowest, respectively. GO, gene ontology; CTen, cell type enrichment; dpi, day post-infection

The four contrasts with the largest numbers of DE genes were used to analyze cell types with the online tool CTen with an enrichment score > 2 as the cutoff for significance. Immune response cells were highly enriched: several different types of lymphocytes, whole blood, CD14+ monocytes, CD33+ myeloid cells, bone marrow, and DCs, all of which were detected in the three contrasts: susceptible vs. non-challenged at 5 dpi, susceptible vs. resistant at 5 dpi, and 5 dpi vs. 1 dpi in susceptible birds (Fig. 5)

. The different types of lymphocytes include CD4+ T cells, CD8+ T cells, CD19+ B cells, and CD56+ NK cells. These results suggest that APEC induces the activation of many immune cells in challenged-susceptible birds by 5 dpi. However, in the contrast of challenged-susceptible vs. non-challenged birds at 1 dpi, the cell type enrichment did not show many immune cells, only CD33+ myeloid, bone marrow, and CD14+ monocyte (Fig. 5). This difference over time may indicate an impairment of precursor immune cells in challenged-susceptible birds early after APEC infection.

GOseq was used to interpret DE genes into a meaningful biological context. Using the default settings, GOseq identified many significant GO terms. Figure 5 presents the top 10 significant GO terms in the four contrasts. For the three contrasts: susceptible vs. non-challenged birds at 5 dpi, susceptible vs. resistant birds at 5 dpi, and 5 dpi vs. 1 dpi in susceptible birds, the significant GO terms that were enriched included defense response, leukocyte activation and differentiation, lymphocyte activation, and immune response, which is in strong concordance with the CTen results. However, in the contrast of susceptible vs. non-challenged birds at 1 dpi, the significant GO terms focused on apoptosis, cell death, and immune response.

### Heatmap pathway analysis

**Fig. 6:**
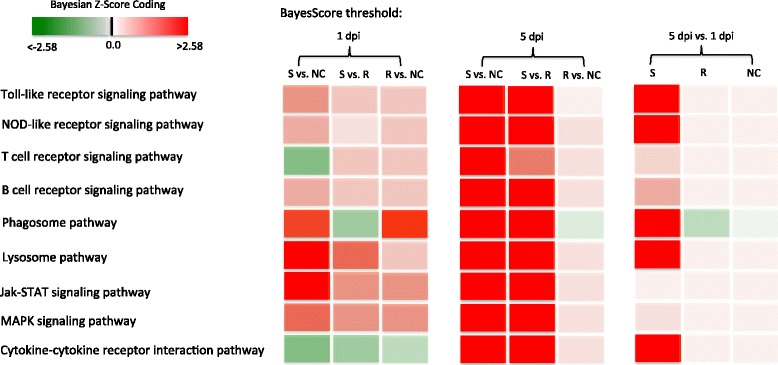
Heatmap comparison of pathway scores for each of the nine two-way contrasts. A gradient color from light to bright red with the score magnitudes indicates different level of induced pathway activity, while a gradient color from light to bright green with the score magnitudes shows different levels of suppressed pathway activity. S, susceptible birds; R, resistant birds; NC, non-challenged birds; dpi, day post-infection

Many canonical pathways were identified as significant in the nine contrasts with FDR controlled at 0.05. Figure 6 shows a heatmap comparison of pathways in the nine contrasts. We detected nine significant pathways that were related to immune system, signal transduction, signaling molecules and interaction, and transport and catabolism. All nine pathways were strongly and significantly induced at 5 dpi in susceptible vs. non-challenged birds and in susceptible vs. resistant birds (Fig. 6)

. Most of the nine pathways had significant induction states in the same two contrasts on 1 dpi (Fig. 6). Only the T cell receptor signaling pathway, phagosome pathway, and cytokine-cytokine receptor interaction pathway had significant suppression in the contrasts of susceptible vs. non-challenged birds or susceptible vs. resistant birds on 1 dpi (Fig. 6). However, these three pathways appeared to reverse to a more inductive state in the same contrasts on 5 dpi. Interestingly, there were no significant pathways associated with the contrasts of 5 dpi vs. 1 dpi in either resistant birds or in non-challenged birds. However, several immune related pathways, phagosome pathway, lysosome pathway, and cytokine-cytokine receptor interaction pathway were significantly induced in susceptible birds over post-infection time (Fig. 6). These results indicate that, in susceptible birds infected with APEC, the host defenses are increasingly induced over time. Moreover, APEC-infected susceptible birds express unique biosignatures compared to resistant birds.

### Quantitative PCR validation for RNAseq results

Quantitative PCR (qPCR) was utilized to validate results of fifteen significant immune-related genes from the RNAseq study (FDR < 0.05): ADA, BLNK, CD3D, CD28, CD40, CD3Z, IFNG, IL1, IL7, IL8, IL18, LIG4, MD2, NOD1, and TLR4. Moreover, all the validated DE genes that we selected had high expression (Additional file 1: Table S2). The qPCR validation was carried out on five contrasts that had the largest numbers of DE genes in RNAseq: susceptible vs. non-challenged birds at 5 dpi, susceptible vs. resistant birds at 5 dpi, 5 dpi vs. 1 dpi in susceptible birds, susceptible vs. non-challenged birds at 1 dpi, and susceptible vs. resistant birds at 1 dpi. Results from qPCR are generally similar to those of RNAseq in both the direction of fold change and significance (Table 2).

**Table 2:** Quantitative PCR validation

Gene	Contrast	qPCR	RNA-seq
ADA	Susceptible vs. Non-challenged birds at 5 dpi	−15.66**	−3.92**
	Susceptible vs. Resistant birds at 5 dpi	−18.99**	−3.39**
	5 dpi vs. 1 dpi in susceptible birds	−9.29**	−2.50**
BLNK	Susceptible vs. Non-challenged birds at 5 dpi	+3.11*	+2.50**
	Susceptible vs. Resistant birds at 5 dpi	+4.71**	+2.22*
	5 dpi vs. 1 dpi in susceptible birds	+3.85**	+2.19**
CD3D	Susceptible vs. Non-challenged birds at 1 dpi	−2.36**	−1.67**
CD28	Susceptible vs. Non-challenged birds at 5 dpi	+7.17**	+2.27*
CD40	Susceptible vs. Non-challenged birds at 5 dpi	+4.40**	+2.93**
	Susceptible vs. Resistant birds at 5 dpi	+2.44**	+2.25**
	5 dpi vs. 1 dpi in susceptible birds	+2.25*	+1.91**
CD3Z	Susceptible vs. Non-challenged birds at 1 dpi	−1.91	−1.77**
	Susceptible vs. Resistant birds at 5 dpi	−7.05*	−2.11**
IFNG	Susceptible vs. Non-challenged birds at 1 dpi	−2.19**	−1.71*
IL1	Susceptible vs. Non-challenged birds at 5 dpi	+2.41*	+5.21**
IL7	Susceptible vs. Non-challenged birds at 5 dpi	−3.38**	−4.69**
	Susceptible birds vs. Resistant birds at 5 dpi	−1.96*	−3.36*
	5 dpi vs. 1 dpi in susceptible birds	+2.72*	+5.43**
IL8	Susceptible vs. Non-challenged birds at 5 dpi	+6.70**	+6.06**
	Susceptible vs. Resistant birds at 5 dpi	+2.69*	+2.72**
	5 dpi vs. 1 dpi in susceptible birds	+3.89*	+2.37*
IL18	Susceptible vs. Non-challenged birds at 5 dpi	+6.47**	+3.97**
	Susceptible vs. Resistant birds at 5 dpi	+2.40*	+2.82**
LIG4	Susceptible vs. Non-challenged birds at 5 dpi	−6.14**	−1.57*
MD2	Susceptible vs. Non-challenged birds at 5 dpi	+2.39**	+3.10**
	Susceptible vs. Resistant birds at 5 dpi	+2.26**	+2.55**
	5 dpi vs. 1 dpi in susceptible birds	+2.16**	+1.83**
NOD1	Susceptible vs. Non-challenged birds at 5 dpi	+2.77*	+2.17**
	Susceptible vs. Resistant birds at 5 dpi	+5.36**	+1.97**
TLR4	Susceptible vs. Non-challenged birds at 5 dpi	+3.26**	+2.77**
	Susceptible vs. Resistant birds at 5 dpi	+2.53*	+2.35**
	5 dpi vs. 1 dpi in susceptible birds	+2.75*	+1.89**

Note: Fold change between contrasts presented in third and fourth column. + values indicate higher expression in the first group, − values indicate higher expression in the second group. ** is *P* value < 0.01 in qPCR or FDR < 0.01 in RNA-seq; * is *P* value < 0.05 in qPCR or FDR < 0.05 in RNA-seq.

## Discussion

This study used a novel experimental design to enable the study of challenged birds with two extreme pathology levels: severe lesions (susceptible) and mild lesions (resistant), with the aim to elucidate resistance and susceptibility mechanisms. This design, therefore, is more comprehensive than previous experiments with other avian pathogens that only assessed the contrast of non-challenged with challenged birds [27, 28]. Figure 2 illustrates the wide distribution of lesion score (0–7) phenotypes in the challenged group. The PCA results (Fig. 3) further validate the concept that the bone marrow transcriptome response of APEC-challenged birds that are susceptible (severe lesion) is very distinct from those that are resistant (mild lesion). Large numbers of DE genes were detected in the four contrasts: susceptible vs. non-challenged at 5 dpi, susceptible vs. resistant at 5 dpi, 5 vs. 1 dpi in susceptible birds, and susceptible vs. non-challenged at 1 dpi. To determine the populations of specific particular cell types that responded to the differences in transcriptional activity, the CTen database information was used to detect the cell types for these four contrasts. The CTen database likely misses the identification of some cell types in the current study, because it is based on mammalian (mouse and human) tissues and cell types. However, because 60 % of chicken genes correspond to a similar human gene [29], the mammalian data in the CTen can serve as an initial reference to identify specific cell types that respond to APEC infection in birds. Large numbers of DE genes were enriched in immune related cells, which corresponds to monocytes, DCs, CD4+/CD8+ T cells, B cells, and NK cells migration to the site of APEC infection. The cell type enrichment, GO term, and pathways analysis are all consistent, thus confirming the results of each individual analysis.

DCs, which are efficient antigen presenting cells (APCs), play a vital role in both innate and adaptive immune response. Based on CTen results, 267 (75.28 % up-regulated), 173 (66.47 % up-regulated), and 167 (82.63 % up-regulated) significant DE genes were detected in DCs in the contrasts of susceptible vs. non-challenged birds at 5 dpi, susceptible vs. resistant birds at 5 dpi, and susceptible birds at 5 dpi vs. 1 dpi, respectively. Environmental stimuli and pathogens can have a major effect on the functions and maturation of DCs [30–32]. Mature DCs that are promoted by toll like receptor (TLR) ligand binding have the ability to drive naive T cells to become effector T cells (T helper 1 and 2) [33, 34].

Monocytes were significantly changed in current study, with 269 (81.78 % up-regulated), 169 (75.15 % up-regulated), and 177 (90.40 % up-regulated) significant DE genes in susceptible vs. non-challenged birds at 5 dpi, susceptible vs. resistant birds at 5 dpi, and susceptible birds at 5 dpi vs. 1 dpi, respectively. Monocytes can differentiate into DCs and macrophages [35–37]. Macrophages have a relative long life-span before they remove the pathogens invasion. The functions of macrophages include phagocytosis of foreign particles, secretion of enzymes and oxidative metabolites, cytokine production, APC, and opsonization [38–40]. Macrophages can produce a variety of cytokines involved in the pro-inflammatory response like IL1, IL6, and TNFα [41, 42]. Also, macrophages have an important role as APC in activating the immune response of T cells.

NK cells, a third lymphoid lineage and thymus-independent, have many similar characteristics with cytotoxic T cells; they both respond against a wide variety of pathogens by production of a serine protease and a pore-forming protein [43, 44]. In the current study, 246 (74.39 % up-regulated), 157 (63.69 % up-regulated), and 133 (82.71 % up-regulated) DE genes were involved in NK cells in the contrasts of susceptible vs. non-challenged birds at 5 dpi, of susceptible vs. resistant birds at 5 dpi, and of 5 dpi vs. 1 dpi in susceptible birds, respectively. The NK cells play a key role in host primary defense and the homeostasis of normal tissues as part of the innate immune system [45]. Taking the results on bone marrow NK cells, macrophages and DCs collectively, there is significant activation of the myeloid cells of the innate immune system occurring soon after APEC infection.

**Fig. 7:**
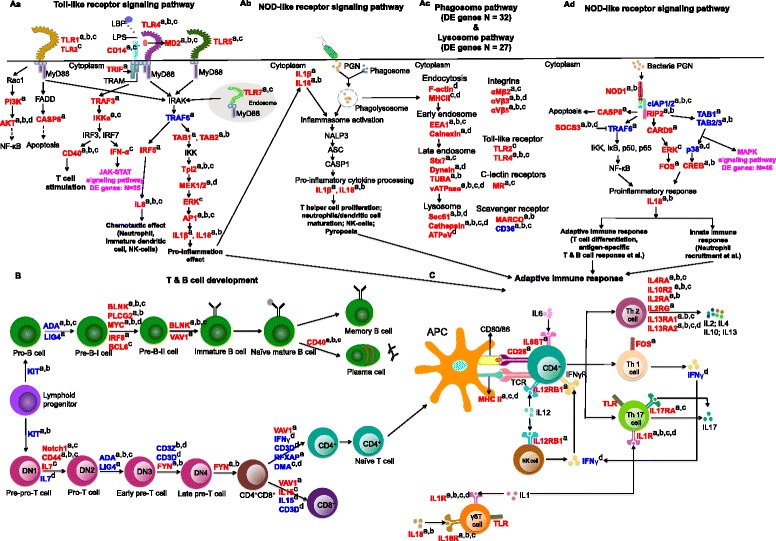
Dynamic differentially expressed (DE) genes were involved in T and B cell development as well as innate and adaptive immune response. Aa, Ab, and Ac were innate immune response including toll-like receptor signaling pathway, NOD-like receptor signaling pathway, phagosome and lysosome pathway. B indicates the process of B and T lymphocytes lymphopoiesis and the regulation genes in each step. C represents adaptive immune response under APEC infection. Genes in blue color were down-regulated while genes in red color were up-regulated. The pink color indicates significantly changed pathways that were not discussed here. The lowercase a, b, c, d indicate different contrasts: a, day 5 susceptible birds vs. day 5 non-challenged birds; b, day 5 susceptible birds vs. day 5 resistant birds; c, day 5 susceptible birds vs. day 1 susceptible birds; d: day 1 susceptible birds vs. day 1 non-challenged birds. LBP, lipopolysaccharide binding protein; LPS, lipopolysaccharide; PGN, peptidoglycan; N, number

In addition to the immune cells, many innate immune response signaling pathways, including TLR- and NOD-like receptors, were significantly changed. Based on chicken KEGG pathways (gga04620 and gga04621) and the bone marrow transcriptome data from this APEC-challenge study, we modelled the detailed interaction of genes in the bone marrow transcriptome in the toll-like receptor and NOD-like receptor signaling pathways (Fig. 7Aa and Ab)

.

TLRs respond to bacterial peptidoglycan (PGN) components in the extracellular environment by triggering production of pro-IL1β and pro-IL18, resulting in intracellular inflammasome activation and leading to NALP3 > ASC > CASP1 > pyroptosis or inflammatory response associated with phagosome and lysosome pathways (Fig. 7Aa, Ab). In the current study, in the important TLR signaling pathway (TLR1/2/4/5/7 > IRAK > TRAF6 > TAB1/2 > IKK > Tpl2 > MEK1/2 > ERK > AP1 > pro-IL1β and IL18 > NALP3 > ASC > CASP1 > IL1β and IL18 cytokines), all genes except for TRAF6 were up-regulated in susceptible vs. non-challenged birds at 5 dpi (Fig. 7Aa). These results indicate that the innate immune response is highly activated in susceptible birds. Similar to the susceptible vs. non-challenged contrast at 5 dpi, the contrasts of susceptible birds at 5 vs. 1 dpi and of susceptible vs. resistant birds at 5 dpi also had higher expression of many of the same genes (Fig. 7Aa). Other signaling pathways were also detected, including TLR1/2 > FADD > CASP8 > apoptosis and TLR4 > TRIF > TRAF3 > IKKe > IRF3/7 > IFNα > JAK-STAT signaling pathway. The DE genes in the above pathways were up-regulated in susceptible vs. non-challenged birds at 5 dpi, and at 5 vs. 1 dpi in susceptible birds (Fig. 7Aa). In summary, most genes in this model exhibit increased expression in challenged-susceptible birds compared to challenged-resistant or non-challenged birds at 5 dpi, indicating that susceptible birds have enhanced activation of their innate immune response after APEC infection.

When bacterial PGN components enter into host cells, phagosomes produced by neutrophils or macrophages are activated and the pathway of NOD1 > cIAP1/2 > RIP2 > TAB1/2/3 > ERK/p38 (MAPK signaling pathway) > pro-inflammatory cytokine (IL8) is initiated. Based on our current study’s data and the chicken KEGG database (gga04621), we propose the model in Fig. 7Ab. All the genes of the NOD1 pathway were significantly DE in susceptible vs. non-challenged birds at 5 dpi in the current study. The initial function of cIAP1/2 is to inhibit cell death [46]. TRAF6 is inhibited by SOCS3 [47], which blocks NF-κB signaling and plays a vital role in the TLR signaling pathway [48]. RIP2 also plays a crucial role in TCR signaling, T cell differentiation, and TLR2/3/4 recruitment [49, 50]. In the current study, SOCS3, NOD1, RIP2, CARD9, ERK, FOS, and IL18 were up-regulated, whereas TRAF6 and cIAP1/2 were down-regulated in susceptible vs. non-challenged birds at 5 dpi (Fig. 7Ab). These data indicate that the RIP2 pathway is enhanced whereas the TRAF6 pathway is impaired in bone marrow cells of APEC-infected birds. Collectively, the defense responses of challenged-susceptible birds at 5 dpi are characterized by the induction of multiple innate immune signaling pathways.

Development of T and B cells was also significantly changed during APEC infection. Based on T and B cell development models in mammals and data of the current study, we propose the important genes and cytokines that influence T and B cell status under APEC infection (Fig. 7B). The crucial genes include KIT, Notch1, CD44, IL7, LIG4, ADA, CD3, FYN, and VAV1 [51–58]. Genes Notch1, CD44, FYN, and VAV were up-regulated in susceptible vs. non-challenged birds at 5 dpi, indicating the general trend of increase of expression of key genes in susceptible birds during APEC infection. Similar phenomena were also observed in susceptible vs. resistant birds at 5 dpi and also in 5 dpi vs. 1 dpi in susceptible birds. However, most of the above genes were down-regulated in susceptible vs. non-challenged birds at 1 dpi, suggesting T cell development was largely impaired at the initial APEC infection time in susceptible birds. In summary, the development of T lymphocytes, TCR signaling were impaired in challenged-susceptible birds at 1 dpi, but were enhanced by 5 dpi.

For pre-BCR and BCR signaling in mammals, BLNK, PLCG2, MYC, VAV1, CD40, and BCL6 play a central role in many B-cell transitions [59–63]. Also, BLNK has been reported to be important to BCR signal transduction in chickens [64]. In the current study, all those genes were up-regulated in susceptible vs. non-challenged birds at 5 dpi and also in susceptible vs. resistant birds at 5 dpi. Moreover, IRF8 is a critical transcriptional regulator of B cell lineage specification, commitment, and differentiation in mice [65]. IRF8 was only up-regulated in susceptible vs. non-challenged birds at 5 dpi. Collectively, our data indicate that pre-BCR and BCR signaling are greatly enhanced at 5 dpi in challenged-susceptible birds, compared to non-challenged and challenged-resistant birds.

The adaptive immune response was also activated by cytokines and chemokines produced from the innate immune response (Fig. 7C). APCs, especially DCs, interact with naive T cells through CD80/86, CD28, MHC II, and TCR to produce effector T cells [66, 67]. In the current study, CD28 was more highly expressed in susceptible vs. non-challenged birds at 5 dpi. MHC II (BLB1 and DMA) was more highly expressed in susceptible birds at 5 dpi than 1 dpi, while it was expressed less in susceptible than non-challenged birds at 1 dpi. These data suggest that host adaptive immunity is impaired immediately post-infection, but becomes actively enhanced over time to resist infection. The cytokines, IL1, IL18, IL6, IL12, and IL17, can interact with T cells to resist infection in humans and mice [68, 69]. In the current study, those cytokines or their receptors were expressed more highly in susceptible vs. non-challenged birds at 5 dpi (Fig. 7C). Moreover, IL1R, IL18R, and IL17R were also more highly expressed in susceptible birds at 5 dpi than at 1 dpi. In humans and mice, T helper 2 (Th2) cells produce cytokines IL2, IL4, IL10, and IL13 to induce antibody production [70, 71]. Although these cytokines did not change in gene expression in bone marrow in the current study, their receptors’ genes had increased expression in susceptible vs. non-challenged birds at 5 dpi, in susceptible vs. resistant birds at 5 dpi, and in susceptible birds at 5 dpi vs. 1 dpi (Fig. 7C). In summary, the higher expression of cytokines and their receptors in susceptible birds at 5 dpi indicates that APEC infection results in the extensive activation of adaptive immune response in bone marrow cells over post-challenge time.

Moreover, as we expected, the apoptosis and other cell death mechanisms were also detected in challenged-susceptible birds compared to non-challenged birds in both 1 dpi and 5 dpi. Apoptosis, a major cell death procession, plays an essential role in organism growth and tissue homeostasis [72, 73]. Normally, apoptosis is accompanyed with inflammation and inflammasome to remove the dead cells or abnormal cells [74]. As the susceptible birds had severe lesions, it was reasonable to observe strong apoptosis and cell death in susceptible birds. At 1 dpi, five DE genes were significantly up-regulated in challenged-susceptible birds in apoptosis pathway compared to non-challenged birds: PIK3CD, CYCS, AKT1, IL1RAP, and PIK3R1. Over time post-infection, apoptosis was enhanced in challenged-susceptible birds. Except the above five DE genes, seven DE genes involved in apoptosis pathway were up-regulated in challenged-susceptible birds compared to non-challenged birds at 5 dpi: CASP6, CAPN1, IL1R1, TNFRSF1A, IL1β, CASP8, and TRADD.

All the above discussions were focused on the four contrasts with large numbers of DE genes: susceptible vs. non-challenged birds at 5 dpi, susceptible vs. resistant birds at 5 dpi, 5 dpi vs. 1 dpi in susceptible birds, and susceptible vs. non-challenged birds at 1 dpi. Although we detected small numbers of DE genes in challenged-resistant birds compared to non-challenged birds (N = 189 at 1 dpi and N = 2 at 5 dpi), many significantly changed pathways were also identified at 1 dpi: phagosome, Jak-STAT signaling pathway, and MAPK signaling pathway (Fig. 6). Only CD33+ myeloid cells were detected in resistant vs. non-challenged birds at 1 dpi, indicating innate immune response is the major mechanism for challenged-resistant birds to respond to APEC infection. Compared to challenged-susceptible birds, day 1 challenged-resistant birds only had fifty DE genes (40 up- and 10 down-regulated). However, of those 40 DE genes, 9 were novel genes; 3 genes’ proteins were not characterized; and the other 28 DE genes were highly related to immune function: CD74 molecule (li); cathepsin Z (CTSZ); Ras family (RIN2 and RASGEF1B); immunoglobulin (IGJ); interleukin (IL7 and IL18R1); tumor necrosis factor (TNFRSF21); transforming growth factor (TGFBI); and erythrocyte membrane protein (EPB41L3). Challenged-resistant birds, therefore, enhanced many immune genes expression to resist the early APEC infection.

## Conclusion

This is the first report, to our knowledge, examining the role of bone marrow cell gene expression in response to APEC infection in chickens. This transcriptome study provides insight and a genome-level view into the response of cell types and genes involved in the earliest phases of the immune response to APEC infection. Our data indicate a dynamic interaction between the innate and adaptive immune responses to APEC infection in susceptible birds, providing flexibility and redundancy in the host’s induction of cytokines and chemokines. Additionally, B cell and T cell development are also extensively affected by APEC infection in challenged-susceptible birds, resulting in drastic host impairment in early response to infection. This impairment of the early response may cause the delayed initiation of the cytokine response, resulting in the greater level of pathology (susceptibility to disease) in these birds. The DE genes related to immune response interaction exert their function in a highly coordinated fashion where multiple pathways are involved in T and B cell development, differentiation, proliferation and maturation in bone marrow. As post-infection time progressed, bone marrow cells of challenged-susceptible birds actively triggered different facets of the immune response. The transcriptomic profile of challenged-resistant birds suggests an immune system that differs only slightly from that of non-challenged birds, perhaps because the challenged-resistant phenotype has little need to activate the immune system at a high level to control APEC-induced disease. In contrast, in the challenged-susceptible birds, the DE genes in the immune cells and the identified signaling models are representative of activation and resolution of infection at both assayed post-infection days. The present study sheds light on the genomic modulation of the immune response against APEC infection in chickens. By contrasting the response of challenged-resistant vs. challenged-susceptible phenotypes, in addition to challenged vs. non-challenged birds, this study also builds a foundation for identifying host genetic variation that may be manipulated to enhance resistance to infection and colibacillosis.

## Additional file

Additional file 1: Table S1. Primers sequence for qPCR validation. Table S2. Quantitative PCR validation. (XLSX 44 kb)
